# Carbon Fabric Decorated with In-Situ Grown Silver Nanoparticles in Epoxy Composite for Enhanced Performance

**DOI:** 10.3390/nano12223986

**Published:** 2022-11-12

**Authors:** Meghashree Padhan, Umesh Marathe, Jayashree Bijwe

**Affiliations:** 1 Centre for Automotive Research and Tribology, Indian Institute of Technology, Delhi 110016, India; 2 Industrial Tribology, Machine Dynamics and Maintenance Engineering Centre, Indian Institute of Technology, Delhi 110016, India

**Keywords:** silver nanoparticles, carbon fabric, epoxy, nano-composites

## Abstract

The current study focuses on studying the effect of reinforcement of carbon fabric (CF) decorated with in-situ grown silver (Ag) nanoparticles (NPs) on the performance properties of epoxy composite. The Ag NPs were grown on carbon fabric by reducing silver nitrate. The main objective of developing such an innovative reinforcement was to improve thermal conductivity, interlaminar strength, and tribological properties of CF-epoxy composites. The growth of NPs on the surface of CF was confirmed through scanning electron microscopy (SEM), energy dispersive X-Ray spectroscopy (EDAS), X-ray photoelectron spectroscopy (XPS), and X-ray diffraction studies. The development of composites was conducted by the impregnation method, followed by compression molding. It was observed that in-situ growth of Ag NPs enhanced thermal conductivity by 40%, enhanced inter-laminar shear strength by 70%, enhanced wear resistance by 95%, and reduced the friction coefficient by 35% in comparison to untreated CF.

## 1. Introduction

Carbon fiber reinforced polymer composites have been applied in diverse fields, such as automotive, aviation, aerospace, fuel cell, turbomachinery, antistatic and electromagnetic shielding, compressed gas storage, transportation, and other related fields, by virtue of their unique properties, including high specific strength, stiffness, thermal stability, self-lubricity, and thermal conductivity. For such polymer composites, the fiber-matrix interface is a critical parameter to performance properties. The surface of carbon fibers, being smooth, chemically inert, and hydrophobic in nature, leads to a weak interaction with polymeric chains. Hence, surface modification of carbon fibers by various techniques (dry, wet, and multiscale), and treatments with chemicals, plasma, high energy irradiation, electrochemical deposition, etc. (excluding the grafting of carbon nanotubes (CNTs), graphene, or some nanoparticles (NPs) etc.), have been successfully explored in the past few decades [1,2,3,4,5,6,7,8,9,10,11,12,13,14,15,16,17,18,19,20,21]. An exhaustive literature survey showed that NPs at the interface greatly improve properties, and the technique has been increasingly explored for strengthening the interface [16,22]. The treatment with YbF_3_ was effective at the optimum dose of 0.3 wt.%. A higher dose of NPs led to agglomeration, reducing the benefits. It is quite challenging to either retain or enhance the benefits by increasing the dose of NPs without agglomeration. The present work addresses this issue, and the results are discussed in the subsequent sections.

Metallic NPs on the surface offer the additional advantage of imparting higher thermal conductivity (TC) and electrical conductivity (EC). Liu and Kumar [23] reviewed the current advancements in carbon-fiber structure, fabrication, and properties in detail, including incorporating carbon nanotubes (CNTs) in the precursor fiber to improve the mechanical properties. Thostenson et al. [24] studied CNTs-carbon fiber, hybrid fibers based on single-fiber epoxy composites. It was observed that CNTs at the fiber-matrix interface improved composites’ interfacial shear strength (IFSS-Interfacial shear strength representing the load transfer efficiency between the fiber and the resin). Karakassides et al. [25] reported a novel nano-reinforced interface by growing radially aligned graphene nano-flakes on carbon fibers using a one-step microwave plasma-enhanced chemical vapor deposition technique, and using them in epoxy-based composites. Significant improvements in the performance of fibers (28% in tensile strength and 157% in electrochemical capacitance) and in composites (101% in IFSS and 60% in EC) were reported. Lin et al. [20] studied a novel nanocomposite with zinc oxide (ZnO) nanowires grown on the surface of the carbon fibers for an enhanced interface for epoxy-based composites. IFSS increased by 113% and ILSS (interlaminar shear strength) increased by 38%. Advanced research recommends the growth of metallic NPs on the surface of carbon fibers, which impart additional advantages of increased TC, EC, and ILSS. Wang et al. [26] studied epoxy composites reinforced with carbon fibers modified with silver NPs deposited by an electrochemical deposition method, which led to an increase in IFSS by 57% and ILSS by 27%. The TC of the fibers also significantly improved. However, the tribo-behavior of such composites has not been investigated. Although some work has been reported on the in-situ grown NPs of Ag [13,26,27,28] on carbon fiber surfaces, little has been reported on their impact on the various performance properties of composites, except for ILSS.

This study planned to grow Ag NPs in situ on the surface of carbon fabric (CF) and use this as a reinforcement for PAEK (Polyaryletherketone) composite by compression molding at 400 °C. The aim was to strengthen the interface and improve thermal conductivity (TC). Initial investigations showed contrasting results (TC decreased by 25%, and the coefficient of friction (µ) increased by 37% compared to the composite with untreated CF). This was attributed to the fact that Ag NPs were oxidized to Ag_2_O around 150 °C during high temperature (400 °C) molding, and Ag_2_O NPs increased abrasion and µ, decreasing the TC. Consequently, it was decided to use epoxy as a matrix that easily cures at room temperature. The investigations are presented in the subsequent sections.

## 2. Materials and Methodology

### 2.1. Materials

The supplier’s data for epoxy resin (medium viscosity at 25 °C-300–700 (mPa s) with a long pot life and a glass transition temperature (T_g_) of 75–85 °C) are provided in Table 1 [29]. The resin is recommended for small to large composite components by Resin Transfer Moulding, Resin Infusion (RI), and hand lay-up. The details of the carbon fabric are given in Table 2. Silver nitrate (AgNO_3_) (MW 169.87) and sodium hydroxide (NaOH) pellets (MW −39.95) were purchased from Thermo-Fisher Scientific India Pvt. Ltd (Mumbai, India). Sodium citrate tribasic hydrate (C_6_H_5_O_7_Na_3_·2H_2_O) (MW 294.10) was obtained from Sisco Research Laboratories Pvt. Ltd., Mumbai, India. Sodium borohydride (NaBH_4_) (MW 37.83) was procured from Leonid Chemicals India Ltd., Bangalore, India.

### 2.2. Methodology

#### 2.2.1. In-Situ Growth of Silver NPs on the Surface of Carbon Fabric

**Figure 1:**
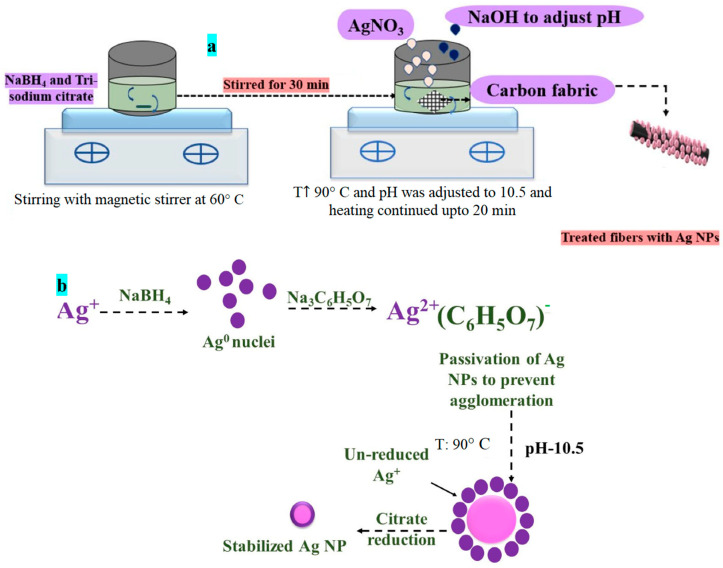
(**a**) Schematic for the process and (**b**) in situ growth of Ag-NPs -mechanism involved.

Agnihotri et al. [32] described a process for synthesizing Ag NPs by reducing silver nitrate, and the same process has been followed in the current work. The primary reductant was sodium borohydride (NaBH_4_), and the secondary reductant and stabilizing agent was trisodium citrate (TSC). Freshly prepared aqueous solutions (1 mM NaBH_4_ and 3.5 mM TSC) were mixed and heated at 60 °C for half an hour in the dark with vigorous stirring. After dipping fabric plies in the solution for half an hour, a freshly prepared solution of 1 mM silver nitrate (AgNO_3_) was added dropwise, followed by increasing the temperature to 90 °C. The pH of the solution was adjusted to 10.5 by adding 0.1 M sodium hydroxide (NaOH), and heating was continued for another 20 min until a color change was observed. The heating was turned off, and the carbon fabric was thoroughly washed with water, followed by oven drying. The process is described in Figure 1a. The reduction occurred in two stages, as shown in Figure 1b.

- NaBH_4_ initiates the reduction of silver cations to form silver nuclei in the first stage. Silver NPs formed at this stage were later involved in the growth process.
- In the second stage, TSC was used to further reduce unconverted Ag ions at 90 °C and 10.5 pH. At low temperatures, the TSC primarily acts as a passivate for the NPs, preventing them from aggregating.
- In the two-stage co-reduction approach, an adequate NaBH_4_ to TSC ratio is a critical controlling parameter for nucleation and growth activities. The objective was to grow identical-sized NPs uniformly dispersed on the surface of carbon fibers.

#### 2.2.2. Development of Composites

**Figure 2:**
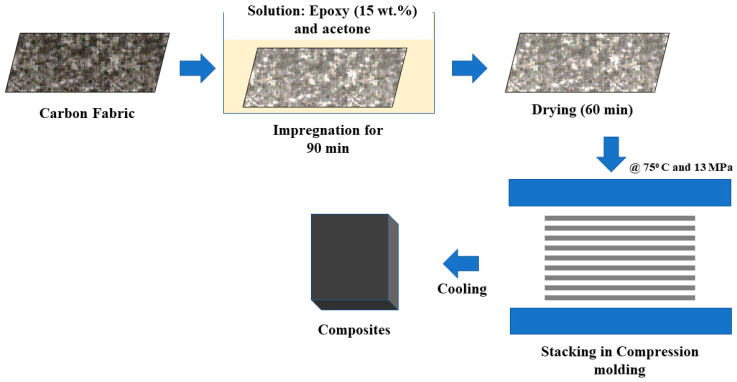
Schematic for the composite fabrication process.

Composites were developed with untreated (U) and treated carbon fabric (T), and epoxy resin using the solution in acetone (15% *w/w* to achieve a 45 wt.% epoxy contents after drying) by impregnation technique, followed by compression molding. Carbon fabric plies (18 cm × 14 cm) were cut from a fabric roll and dipped in the solution for 1.5 h. Plies were carefully removed without disturbing or misaligning the weave, followed by drying for 1 hour at ambient conditions to remove the solvent. PTFE-coated glass fabric was used as a mold release agent, and was placed at the top and bottom of the eight-stacked prepregs. The mold was heated slowly for 1 hour to 75 °C, and then the prepregs were compression molded under a pressure of 13 MPa. Five intermittent breathings were applied to remove any remaining solvent. Under applied pressure, the composites were cooled in ambient conditions. The schematic is shown in Figure 2. The required specimens were cut using a diamond cutter on an Isomat 1000 Precision Saw by Buehler, Leinfelden-Echterdingen, Germany. 

### 2.3. Characterization of Fibers and Composites

#### 2.3.1. Characterization of Fibers

- **Field-emission scanning electron microscopy and Energy Dispersive Spectroscopy (FE-SEM and EDAX)**

The surface of the fibers were analyzed using a JEOL JSM 7800F FE-SEM and energy dispersive X-ray spectroscopy (EDS) (Oxford, UK) to observe the growth of NPs, and elemental mapping to confirm their presence. The EDS dot data were acquired using an EDS detector and EDS system.

- **X-ray diffraction (XRD)**

XRD was used to confirm the presence of Ag NPs on the fibers. The fibers were scanned at a scan rate of 5°/min for two ranges of 10° to 80° using a Rigaku X-ray machine (Tokyo, Japan). The XRD data were analyzed using X’Pert High Score Plus Version 4.9 2020, PANalytical Inc, Westborough, MA, USA, to confirm the presence of Ag NPs.

- **X-ray photoelectron spectroscopy (XPS)**

XPS analyses (XPSO micron ESCA, monochromatic Al K (1486.7 eV) source) were performed to determine the presence of Ag particles and other compounds based on their binding energy.

- **Wettability analysis**

A Kruss goniometer, GmbH (Hamburg, Germany), was used to examine the surfaces of untreated and decorated fibers to determine the contact angle with a water drop. The surface free energy was calculated using the Fowkes method. The volume dropped for the contact angle measurement was 2 µL, and the rate was 0.5 µL/s for water and n-hexane.

#### 2.3.2. Characterization of Composites

- **Physical properties**

The density of the composite was determined using the Archimedes principle on a weighing balance (Mettler Toledo) at room temperature, and the density of the composite was determined using Equation (1):

(1) $$\rho_{c}=\frac{\rho_{liquid}\times W_{air}}{W_{air}-W_{liquid}}$$

where *ρ_c_* is the density of the composite, *ρ_liquid_* is the density of water used as a medium; *W_air_* is the weight of a sample in the air; *W_liquid_* is the weight of a sample fully immersed in water.

- **Thermal conductivity (TC)**

The TC of the disc (50 mm diameter) of composites was measured on a DTC 300 (TA Instruments, New Castle, DE, USA), which operates on the guarded heat flow method principle.

- **Thermal stability by thermogravimetric analysis (TGA)**

The thermal stability of the composites was investigated using a Linseis 1000PT (Selb, Germany) simultaneous thermal analyzer in an air atmosphere at a rate of 10 °C/min from room temperature to 900 °C. Weight loss of around 6–8 mg of the sample was recorded as a function of temperature.

- **Inter-laminar shear strength (ILSS)**

To analyze the fiber-matrix interface, the ILSS of composites was measured as per ASTM D 2344 on a UTM, Instron model 3365 (Norwood, MA, USA), in a three-point bending configuration with a crosshead speed of 5 mm/min and a span length of 26 mm.

- **Tribo-evaluation of composites**

**Figure 3:**
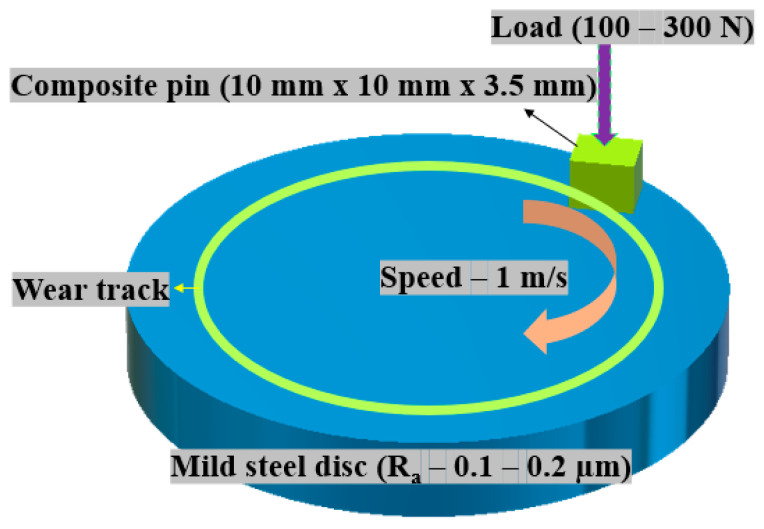
Pin-on-disc configuration used for tribo-evaluation.

In a pin-on-disc configuration, the tribo-performance of composites was evaluated in an adhesive wear mode on CETR (Bruker) UMT-3MT tribometer (Figure 3). A pin of a composite (10 mm × 10 mm × 3.5 mm) slid against the counterface; mild steel disc (Ra—0.1–0.2 m) in a rotary motion. The bedding was initially performed at a load of 50 N, 0.025 m/s to achieve conformal contact. Before and after the experiment, the pin was ultrasonically cleaned in acetone, dried, and weighed. The final weight was determined using a Mettler-Toledo weighing balance with a 0.00001 g accuracy. The load was varied in steps of 100 N, and the speed was maintained at 1 m/s, resulting in a sliding distance of 7200 m in 2 h. Each experiment was repeated twice, and an average of three readings were taken. The following equation was used to determine the specific wear rate (*K*_0_):

(2) $$K_{0}=\frac{\Delta w}{\rho Ld}\;m^{3}/\mathrm{Nm}$$

where Δ*w* is the weight loss of the composite pin, *ρ* is the density of a composite, *L* is the applied load, and *d* is the sliding distance. The coefficient of friction (µ) was recorded automatically by UMT (Bruker, Billerica, MA, USA) (Version 2017) software, and an average value was considered. In every experiment, fresh pins and discs were used.

- **Worn surface analysis**

The worn surfaces of composite pins and transferred films on the discs were gold-coated using a sputter coater (Cressington sputter coater 108, Watford, UK) to observe wear mechanisms with scanning electron microscopy (SEM) (Zeiss EVO MA 10, Jena, Germany) and Bruker EDS.

- **AFM studies**

The surface profile of composite T worn at 300 N was examined with the help of an atomic force microscope (Flex Axiom, Nano surf, Liestal, Switzerland) in a contact mode. Lateral force microscopy (LFM) was also used to map the friction involved with different constituents on the selected contour of the composites, along with a topographic scan. The acquired signals were processed with the help of Gwyddion software (Version 2.62) by Department of Nanometrology, Czech Metrology Institute..

## 3. Results and Discussions

### 3.1. Treated Fibers

- **SEM and EDAX of fibers**

**Figure 4:**
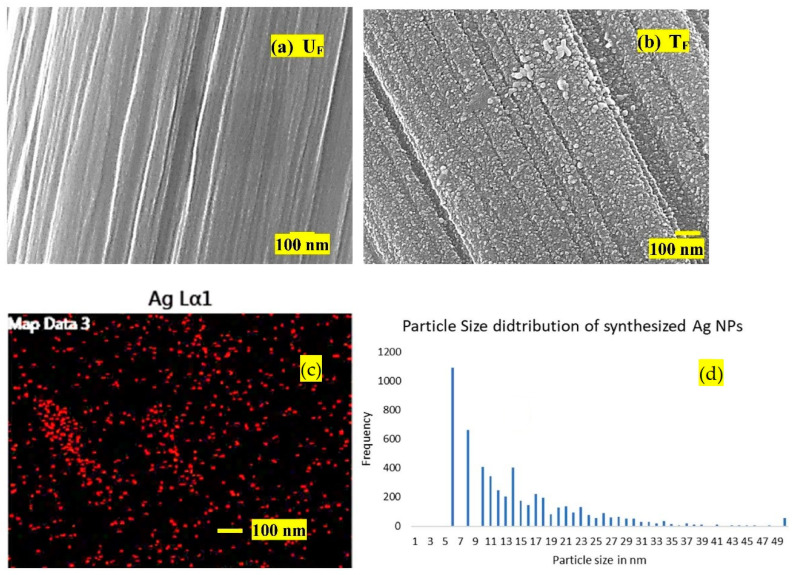
FE-SEM micrographs of (**a**) U_F_ and (**b**) (T_F_): (**c**) EDS data (Ag dot map) for T_F_ and (**d**) particle size distribution for Ag NPs.

FESEM was used to observe the fiber surfaces, and micrographs are shown in Figure 4a,b. Untreated fibers (U_F_) showed very smooth topography, while the surface of treated fibers (T_F_) showed a rough topography and uniformly distributed Ag NPs all over the fiber surface. The NPs appeared spherical and efficiently covered the fabric’s surface. As the particles were grown in situ over the surface of the carbon fibers, they seemed to be de-agglomerated. The treated fibers were simultaneously subjected to elemental analysis by EDAX for the detection of Ag NPs on the surface. The corresponding Ag dot map is presented in Figure 4c. Figure 4d represents the particle size distribution of Ag NPs based on image analysis of FESEM micrographs (Figure 4b). The major portion of particles lay in the range of 10–20 nm.

Carbon/graphite fibers are inert in nature and require surface treatment or compatible sizing agent to promote adhesion between fiber and matrix. The sizing is usually polymer-based (epoxy-based), and also contains antistatic agents, film formers, silane agents, etc. [33]. In the current study, carbon fibers were used and received with a sizing agent. Silver ion (initially) and Ag NPs (later) anchors to the silane sites and epoxy rings. This interaction could be secondary or covalent. During chemical reactions (while generating Ag NPs), crevices may be generated on the surface of precursor fiber. Hence, there could be a component of physical interaction as well.

- **XRD analysis of fibers**

**Figure 5:**
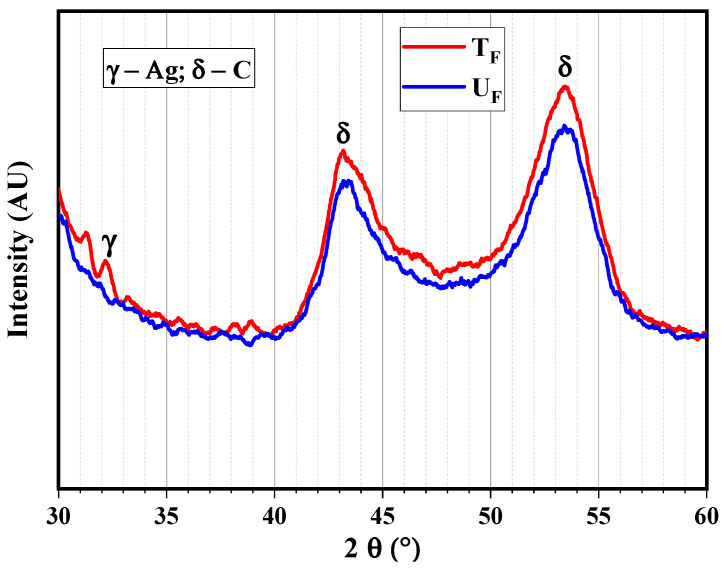
X-ray diffractograms of untreated and treated fibers at scan rate 5°/min for 2θ varying from 10 to 80°.

The XRD diffractograms of untreated (U_F_) and treated (T_F_) fibers are shown in Figure 5.

The peak for Ag was observed at 2θ–32.5°, and the peaks corresponding to carbon are evident from both the diffractograms at 2θ–44.5° and 53.2°.

- **Wettability analysis of fibers**

**Figure 6:**
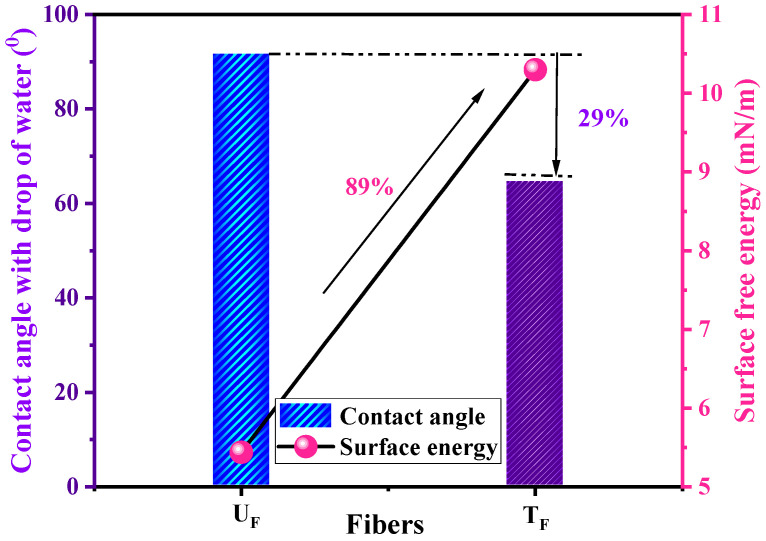
Contact angles and surface energies of untreated (U_F_) and treated (T_F_) fibers.

The goniometry experiment for wettability was performed on the carbon fabric surface; the results are shown in Figure 6. The surface of the untreated fabric showed a higher contact angle (92°), and hence, higher hydrophobicity than the treated fabric with a lower contact angle (65°). The same trend was observed for surface energy, which showed a 89% improvement in the case of T_F_ compared to U_F_. The enhancement can be attributed to uncountable metallic NPs on the surface with very high surface energy.

- **XPS analysis of fibers**

**Figure 7:**
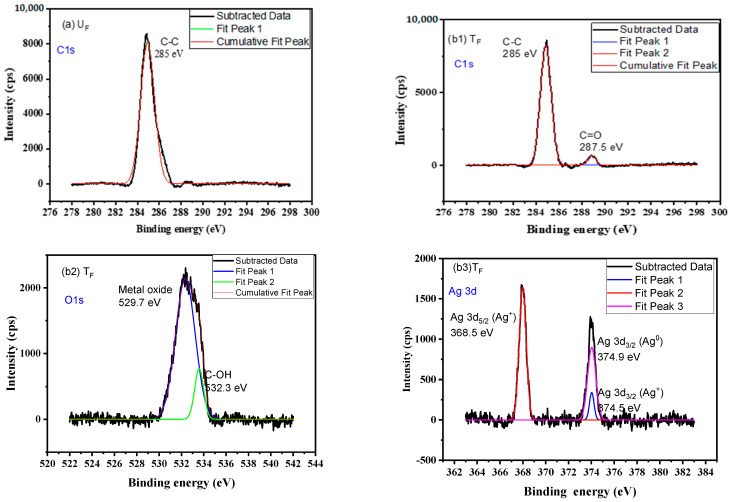
XPS spectra of (**a**) U_F_ for C1s; (**b**) T_F_ with (**b1**) for C1s, (**b2**) for O1s, and (**b3**) for Ag 3d.

Figure 7 shows only C-C bonds corresponding to 285 eV, whereas T_F_ shows C=O (287.5 eV), O-H (531.3 eV), Ag° (374.9 eV), Ag^+^ (374.5 and 368.5 eV), and also the presence of silver oxide (529.7 eV) [34].

It could be concluded that, apart from the growth of silver NPs on the surface of carbon fabric in the case of T_F_, functional groups such as hydroxyl and carbonyl groups were also developed due to chemical reactions by the reducing agent, stabilizer, and NaOH.

### 3.2. Characterization of Composites

#### 3.2.1. Physical Characterization (Density and Void Content)

The density of the treated composite (T) and untreated composite are shown in Table 3. It was observed that the T showed higher density due to NPs of Ag.

#### 3.2.2. Thermogravimetric Analysis of the Composites

**Figure 8:**
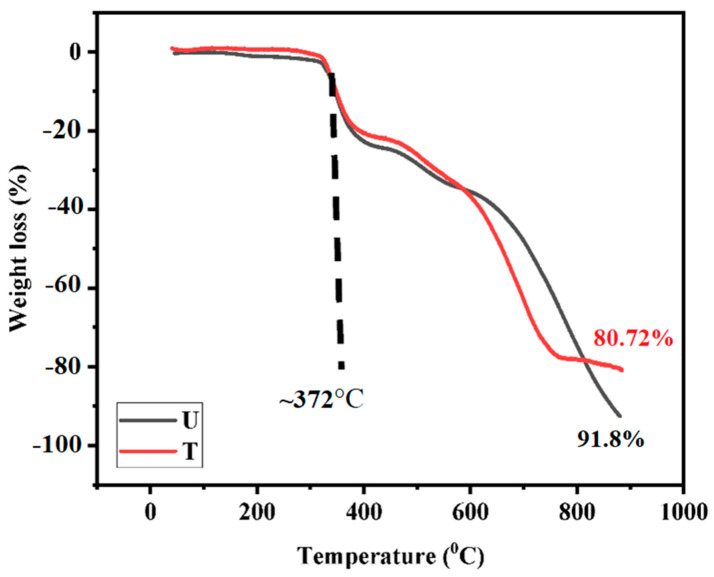
TGA thermograms of composites in air atmosphere at 10 °C/min.

The TGA thermograms of the composites are shown in Figure 8.

Figure 8 depicts the thermogravimetric analysis for the developed composites in an air environment. Epoxy, Ag NPs, and carbon fibers were the major components for composite T, whereas composite U had only two constituents, i.e., epoxy and carbon fiber. It is evident from Figure 8, i.e., TGA curve, that both composites follow similar degradation steps, except in the range of 600–900 °C. The T_10_ (the temperature at which 10 wt.% weight loss was observed) for both composites was observed at ~370 °C due to the initiation of degradation of epoxy in air. Higher temperature behavior was attributed to the presence of silver oxide, which supported the higher yield at the end of the measurement (due to the Ag NPs on carbon fibers [29].

#### 3.2.3. Thermal Conductivity (TC)

**Figure 9:**
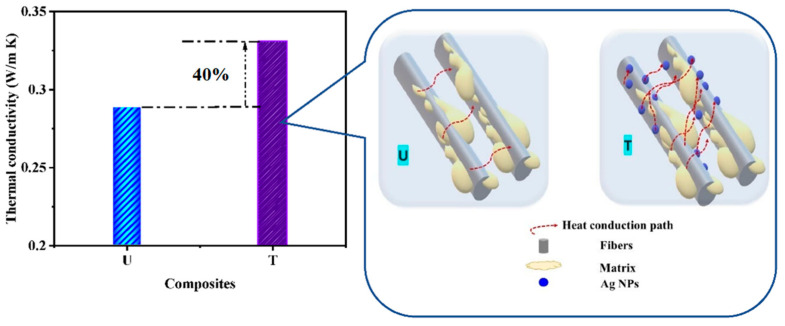
Thermal conductivity of developed composites with their schematic of heat conduction.

The TC of composites is plotted in Figure 9. The matrix, fillers, and concentration primarily control polymer composites’ TC and the microstructure and interfacial bonding between the resin and filler [35]. The continuous carbon fiber structure has inherent advantages in heat conduction. It can directly utilize the moderately high TC of carbon fiber in the axial direction. However, the connectivity between the fibers to further improve the thermal conduction paths in all directions remains a challenge. In this case, Ag particles made a path between the fibers (Figure 9).

Composite T showed a TC of 0.33 W/m K, which was 40% higher than the composite U having a TC of 0.29 W/m K. This is due to Ag NPs attached to the CF surface, which increased the contacts between carbon fibers and epoxy, thereby enhancing the TC. The TC in the case of composite U is due to the heat conduction path by the bonded carbon structure. In contrast, for composite T, the heat conduction path increased because of an enhanced contact area by the NPs, which, in turn, led to a wider passage for heat conduction [35].

#### 3.2.4. Interlaminar Shear Strength of the Composites (ILSS)

**Figure 10:**
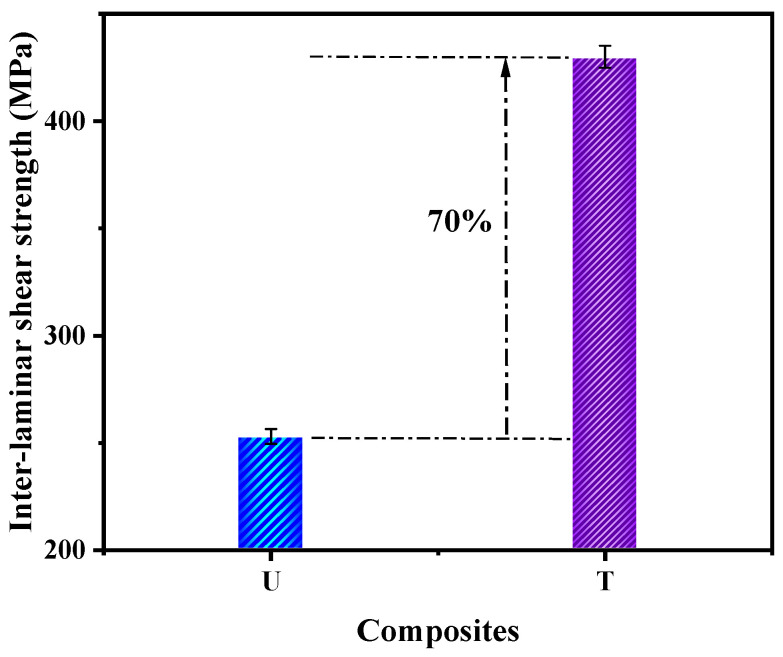
ILSS of composites (crosshead speed 5 mm/min, span length 26 mm).

The ILSS of composites is shown in Figure 10. The composite T showed a 70% enhancement in ILSS (430 MPa) compared to composite U (253 MPa). This was attributed to the presence of Ag NPs on the surface of carbon fibers, which provided a larger surface area for interaction with the matrix. In addition, the treated fibers showed higher surface energy (Figure 6) than the untreated fibers, leading to a stronger interface. The metallic NPs bridge the carbon fibers and the epoxy matrix, enhancing interlaminar shear strength.

#### 3.2.5. Tribo-Evaluation of Composites in Adhesive Wear Mode

**Figure 11:**
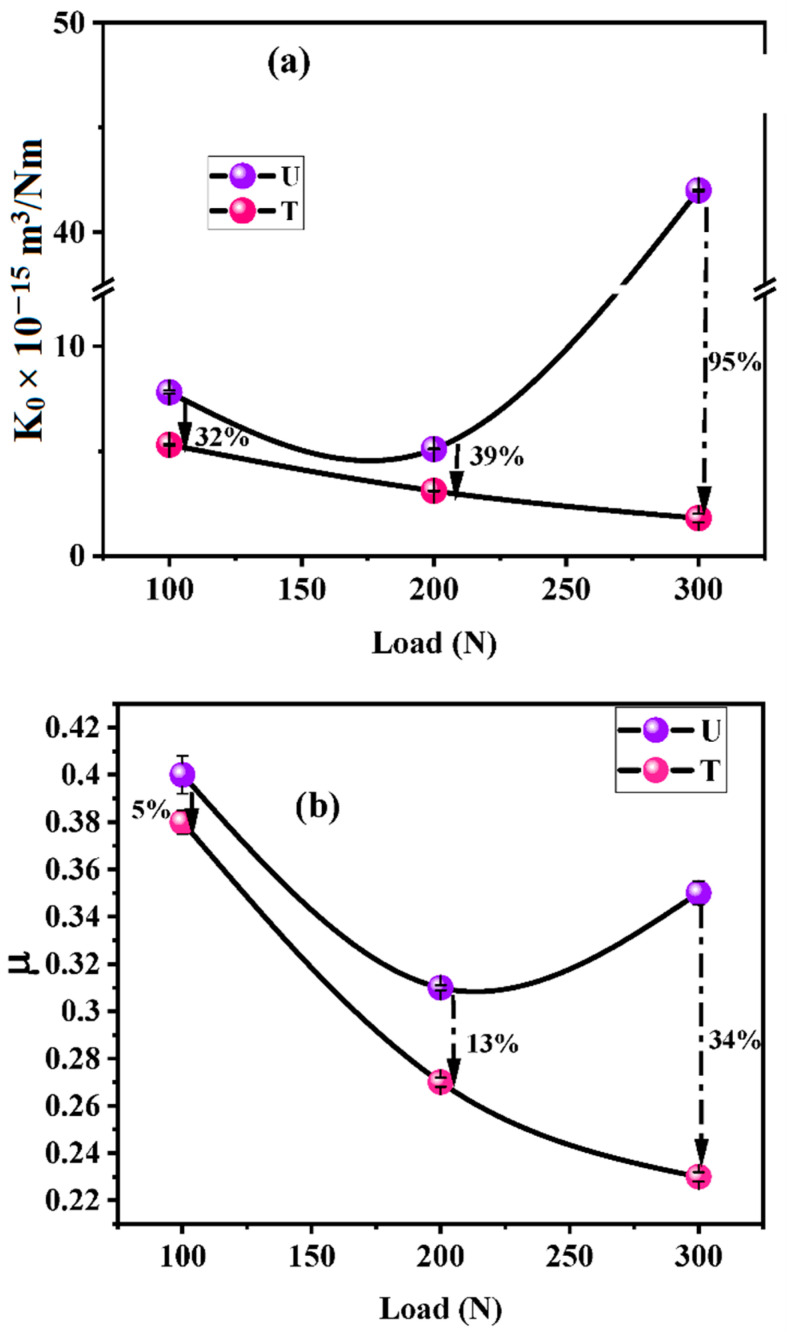
(**a**) Specific wear rate (K_0_) and (**b**) µ for composites as a function of load (Load—100, 200, and 300 N, speed—1 m/s, sliding distance—7200 m, and time—2 h.).

The tribo-performance of the composites was studied by recording the coefficient of friction (µ) and specific wear rate (K_0_), as shown in Figure 11. It was observed from Figure 11 that K_0_ and µ decreased with increasing load for both the composites, as per general trends. When the composite nears the PV_limit_ value, either of the two or both show a sudden rise. In the case of composite U, a sudden rise in both factors was observed. For composite T, not only was friction and wear performance superior to composite U, but the PV_limit_ value also increased by 1 MPa m/s (50%) (the fresh sample was further tested under 350 N. However, it failed after 1 h of sliding with excessive noise and increased wear rate). K_0_ × 10^−15^ m^3^/Nm.

The composite U showed higher K_0_ and µ as compared to composite T for all loads (Table 4).

**Figure 12:**
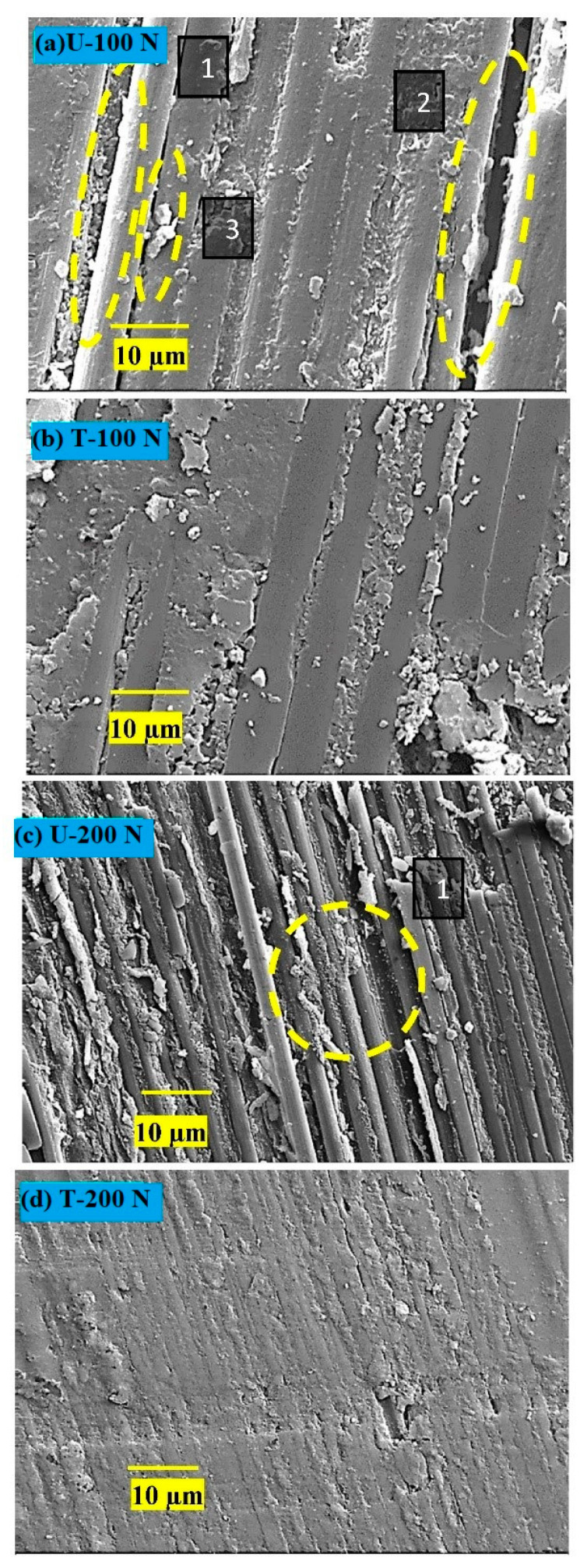
Worn surfaces of composite pins slid at 100 N and 200 N; (**a**) and (**c**) for composite U; (**b**) and (**d**) for composite T. (1) Peeled-off fiber during shearing stresses due to weak fiber-matrix interface. Such fibers are vulnerable and removed easily from the surface, leading to cavities and increased fiber-matrix debonding (2). Fiber surfaces are cylindrical, indicating less participation in the wear-thinning process. (3) Pulverization of carbon fibers leads to more wear.

Wear of fiber/fabric-reinforced composites occurs because of successive events of fiber failure. Fibers are responsible for enhancing wear resistance (W_R_) and reducing friction in case they are carbon fibers, due to their self-lubricating nature. Due to shearing stresses, the fiber-matrix debonding process is initiated during sliding. Debonded fibers are easily cut, and fibrous wear debris is dug out in successive sliding. This debris then starts controlling friction as a vicious cycle. If the fiber-matrix interface is robust, these processes get delayed, and fibers are worn lengthwise instead of debonding and micro-cutting. This leads to a higher W_R_ of a composite. In the case of composite T, the fiber-matrix interface was relatively stronger due to Ag NPs at the interface, as evident from Figure 12. The ILSS of composite T is 70% higher than that of composite U. The higher thermal conductivity (40%) of composite T than composite U is also responsible for superior tribo-performance. The frictional heat generated at the fiber-matrix interface is efficiently conducted away due to Ag NPs, reducing the severity of fiber-matrix debonding. NPs are well known for their high surface energy, high surface-volume ratio, better interaction with the matrix, and better bonding, leading to less wear of composite T.

With increasing load, the polymers and fibers tend to more quickly transfer a beneficial transfer film with better quality onto the disc, changing the contact from polymer against metal to polymer against a polymer. This, in turn, leads to lower µ and K_0_. In the case of composite T, the reduction in friction could be due to the Ag particles, which are known as solid lubricants, thereby gaining additional lubricity [36]. It was thought necessary to investigate the lubricating properties of Ag NPs, and hence, AFM studies were conducted and are presented in the subsequent section.

#### 3.2.6. Worn Surface Analysis

##### SEM Studies

The surfaces of composite pins worn under two loads (100 and 200 N) were examined with SEM; the micrographs are shown in Figure 12. The primary wear mechanisms are marked and labeled as 1, 2, and 3. It appears that the extent of processes due to shearing stresses, viz. increasing fiber-matrix debonding vis-à-vis fiber peeling off from the matrix and rendering them vulnerable for easy pulverization, causing more friction and wear, depended on the load applied and the Ag NP treatment. Higher load resulted in more damage to fibers and increased bonding (Micrographs Figure 12a,c). The wear mechanisms were mild for the treated composite (Micrographs Figure 12b,d). The surfaces were smoother, and the fiber-matrix interface was stronger than that in U. The increase in wear resistance (W_R_) of the composite T can be attributed to:

- Stronger fiber-matrix interface which resisted fiber debonding and pulverization, and ultimately did so easily not succumb to pulverization.
- Wear thinning of longitudinal fibers in a direction parallel to sliding.

In all the cases, extensive damage was observed for composite U. However, fibers remained protected by the matrix in the case of T. For the worn surfaces of composites at 100 N, composite U showed broken fibers, peeled-off fibers leaving behind cavities, and retained a cylindrical shape. In contrast, T showed a few traces of broken fibers, with most fibers protected by a matrix layer. The wear-thinning of fibers resulted in flat surfaces instead of cylindrical ones, and was the main feature supporting the higher wear resistance of composite T.

##### SEM-EDAX Studies on the Films on the Worn Discs

**Figure 13:**
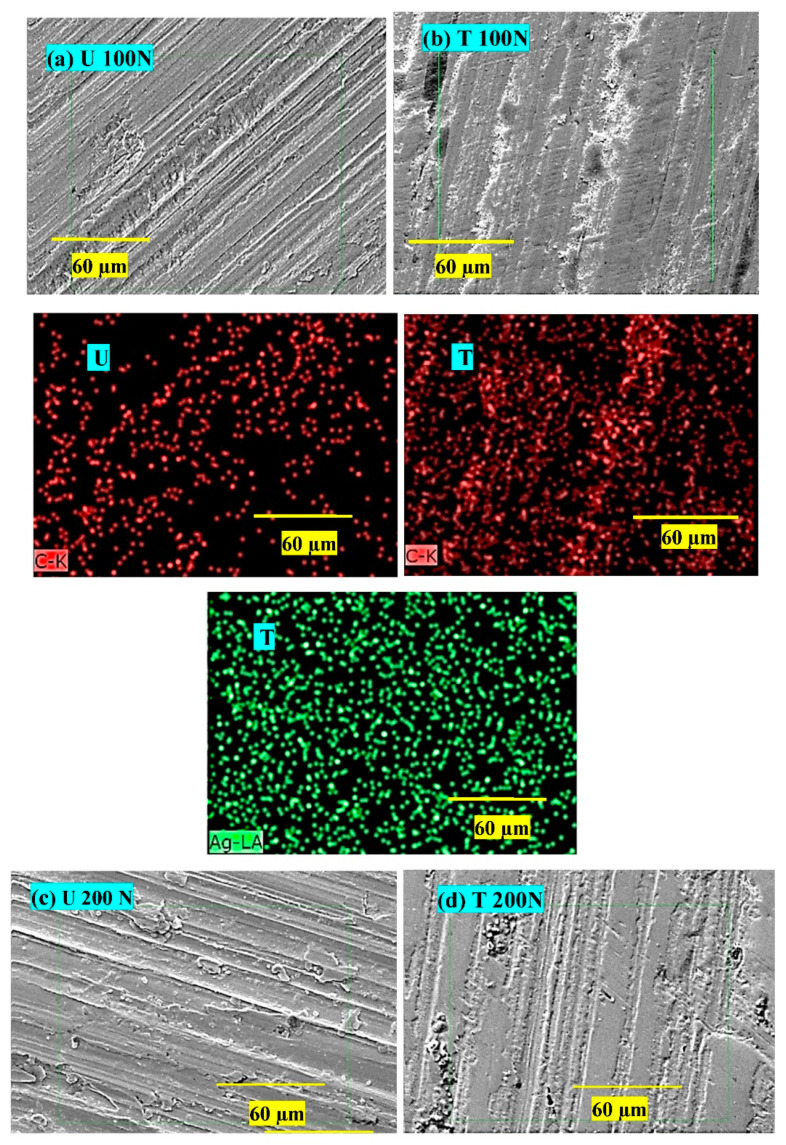

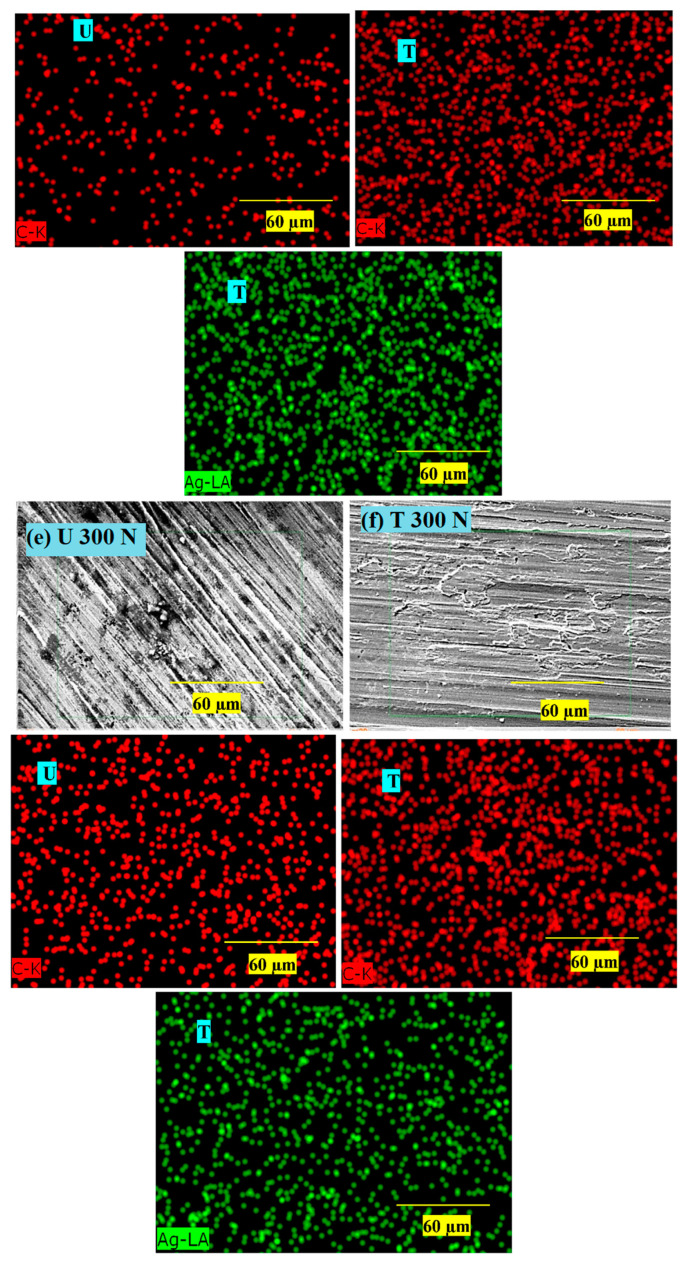
SEM-EDAX dot maps of the wear tracks on the discs worn at 100–300 N against composite U (**a**,**c**,**e**) and composite T (**b**,**d**,**f**). * yellow line indicates the scale and green square indicates the region of interest.

The counterface discs with wear tracks were subjected to elemental analysis by EDAX, and the corresponding dot maps are shown in Figure 13. The left column contains micrographs and a carbon dot map for composite U, while the right one contains those for composite T.

At 100 N (Figure 13a,b), thin film transfer on the disc worn against composite T is more uniform (C dot maps correspond to micrographs; Figure 13a,b), covering almost whole asperities. The transfer film in the case of composite T consists of Ag NPs, as is evident from the Ag dot map; even the carbon density was higher, indicating a coherent film of epoxy, carbon fibers, and Ag. At 200 N (Figure 13c,d), the film transfer quality on both discs was denser and better than that at 100 N; it was thicker and more uniform, especially for the disc worn against composite T. The dot density for carbon and Ag was higher than the earlier density, supporting the heavier transfer on the disc. At 300 N (Figure 13e,f), the film transfer quality on both discs was better than those at 100 N. It was thicker and more uniform, especially for the disc worn against composite T. The dot density for carbon and Ag was higher than the earlier density, supporting the heavier transfer on the disc.

#### 3.2.7. AFM Analysis of the Worn Pin

**Figure 14:**
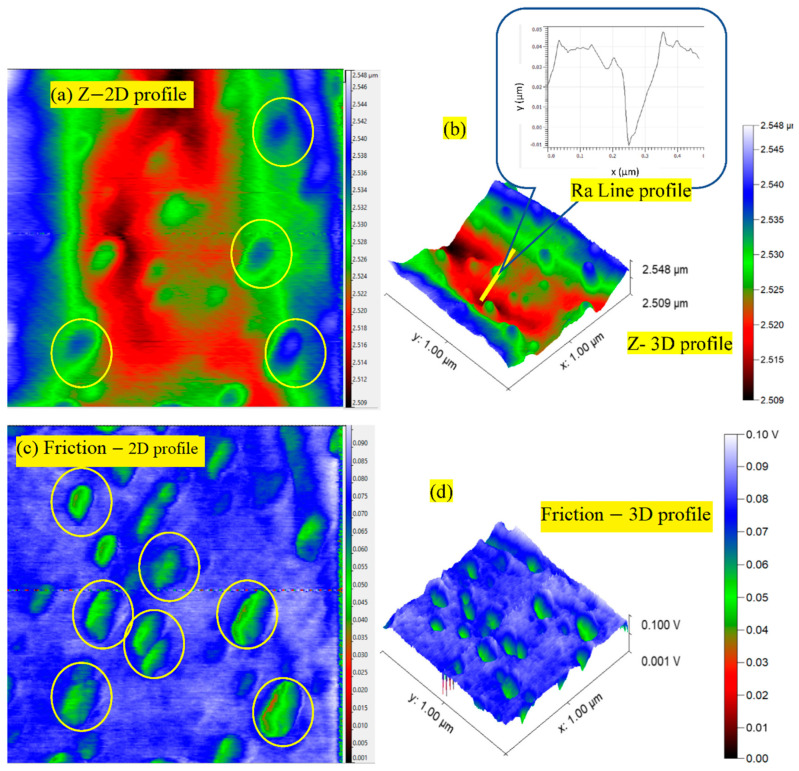
(**a**,**b**) Z-profiles (2D and 3D) and (**c**,**d**) friction profiles in 2D and 3D forms for the worn pin of composite T slid at 300 N, 1 m/s (scanning area of 1 μm × 1 μm).

The surface of composite T (worn under 300 N and 1 m/s speed) was mainly investigated for excellent performance in adhesive wear mode, and to highlight the lubricity of Ag NPs by atomic force and lateral force microscopy (Figure 14). In Figure 14a, worn surface topography with varying roughness can be observed. The rougher spots could be attributed to the wear debris produced (and then embedded) due to different wear mechanisms like fiber matrix delamination, pull-out, pulverization, etc. High trace for 2D and 3D profiles were found in agreement with SEM micrographs (Figure 14a,b). Moreover, the lateral force microscopy depicts the frictional force traces in Figure 14c,d. Silver, a malleable and soft metal, is known to have low friction [37]. As seen in Figure 14c, the green spots due to domains of Ag NPs show the lowest indicative friction, while the blue domains (due to matrix and fibers) show higher indicative friction. Silver-NPs were initially spherical with an average diameter of 10–20 nm. The AFM image shows rod-shaped clusters of Ag NPs of an average diameter of ~100 nm and a length of ~200–300 nm. Due to friction-induced stress, they appear to be extensively elongated in the sliding direction. For Ag, being excessively malleable, the shearing forces smear off the NPs and form a cluster of larger dimensions.

#### 3.2.8. Wettability Analysis of Wear Tracks 

**Figure 15:**
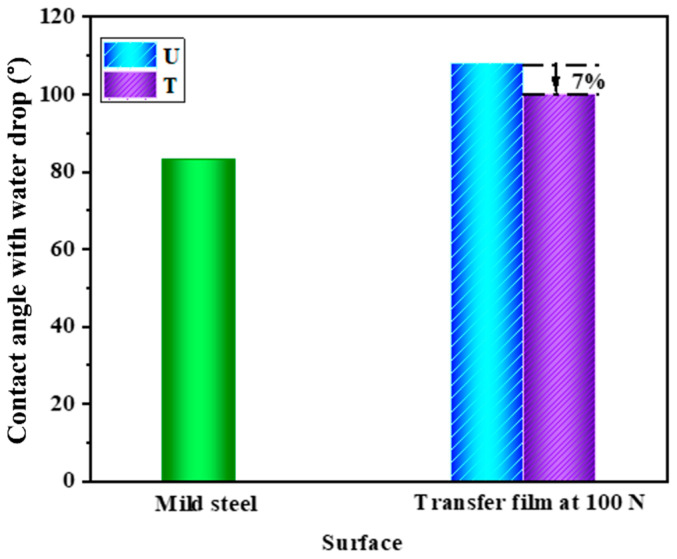
Contact angle on the wear tracks on the discs.

Film on the steel disc was examined for wettability/hydrophobicity, as shown in Figure 15, on a goniometer using a water drop. The fresh surface of the disc showed the lowest contact angle because of the metal’s high wettability/surface energy/hydrophilicity. However, the contact angle increased on the worn disc by composite U, having a transferred film by polymer and fibers. This was due to the hydrophobic nature of the polymeric film, which repelled the water drop. Interestingly, a decrease in water contact angle for the transferred film by composite T was observed compared to composite U. This may be due to the additional presence of film produced by Ag NPs, which has a metallic character, and hence, increased hydrophilicity compared to the polymer.

## 4. Conclusions

**Figure 16:**
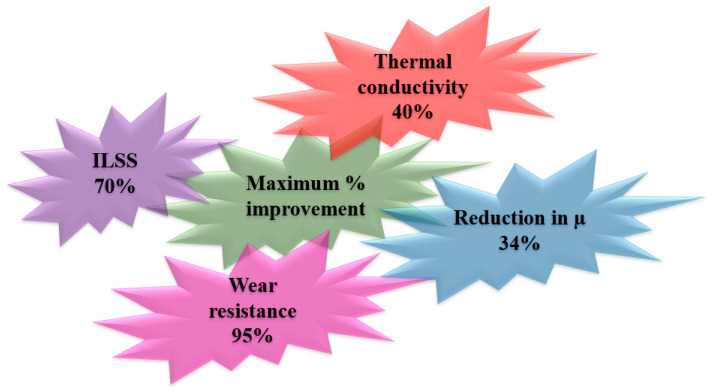
Performance enhancement due to in-situ grown Ag NPs on carbon fabric.

The present study explored the effect of in-situ grown Ag NPs on the surface of carbon fabric to enhance the performance properties of the carbon-fabric reinforced epoxy composite. It was observed that the novel concept of in-situ grown NPs on carbon fabric successfully improved the properties manifold as they grew in de-agglomerated form. A significant improvement in the performance properties of carbon-fabric (55 wt.%)-reinforced epoxy composite by strengthening the interface was as follows (Figure 16).

It was also concluded that the process has significant potential for enhancing the performance of composites. In the future, the process of decorating the interface with Ag NPs can be optimized by selecting different doses of reactants for various durations. Since carbon-fabric-reinforced epoxy composites are well suited for structural applications in the automotive and aircraft industries, these composites can be explored further.

## Figures and Tables

**Table 1:** (a) Details of epoxy resin [29,30].

Properties	Values
Trade name	Lapox ARL-125
Supplier	Atul Polymers India
Density (g/cc)	1.1
T_g_ (°C)	75–80
Curing cycle	70 °C for 8 h
Mixing ratio	100:32

**Table 2:** Details of carbon fabric [31].

Properties	Values
Supplier	Fiber Glast Ltd., Brookville, OH, USA.
Weave, Area (kg/m^2^)	Twill and 1980
Density (kg/m^3^)	1850
Tow and tex	3K and 22
Denier and count	198 and 26
Thickness (m)	0.0034
Tensile strength (MPa)	0.147
Elongation (%)	1.85

**Table 3:** Properties of developed composites.

Composites	Density (g/cc)
U	1.46
T	1.48

**Table 4:** Comparative performance of composites under varying loads.

Load	µ	K_0_ in the 10^−15^ m^3^/Nm	% Improvement
100 N	U (0.40) > T (0.30);	U (7.81) > T (5.3);	32% in K_0_; 5% in µ
200 N	U (0.31) > T (0.27);	U (5.1) > T (3.1);	39% in K_0_; 13% in µ
300 N	U (0.35) > (0.23)	U (42) >>>> (1.8)	95% in K_0_; 34% in µ

## Data Availability

There is no data to report.
